# An EU Perspective on Biosafety Considerations for Plants Developed by Genome Editing and Other New Genetic Modification Techniques (nGMs)

**DOI:** 10.3389/fbioe.2019.00031

**Published:** 2019-03-05

**Authors:** Michael F. Eckerstorfer, Marion Dolezel, Andreas Heissenberger, Marianne Miklau, Wolfram Reichenbecher, Ricarda A. Steinbrecher, Friedrich Waßmann

**Affiliations:** ^1^Department Landuse & Biosafety, Environment Agency Austria, Vienna, Austria; ^2^Department GMO Regulation, Biosafety, Federal Agency for Nature Conservation, Bonn, Germany; ^3^EcoNexus, Oxford, United Kingdom

**Keywords:** new genetic modification techniques (nGM), genome editing, CRISPR/Cas, plant modification, biosafety, risk assessment

## Abstract

The question whether new genetic modification techniques (nGM) in plant development might result in non-negligible negative effects for the environment and/or health is significant for the discussion concerning their regulation. However, current knowledge to address this issue is limited for most nGMs, particularly for recently developed nGMs, like genome editing, and their newly emerging variations, e.g., base editing. This leads to uncertainties regarding the risk/safety-status of plants which are developed with a broad range of different nGMs, especially genome editing, and other nGMs such as cisgenesis, transgrafting, haploid induction or reverse breeding. A literature survey was conducted to identify plants developed by nGMs which are relevant for future agricultural use. Such nGM plants were analyzed for hazards associated either (i) with their developed traits and their use or (ii) with unintended changes resulting from the nGMs or other methods applied during breeding. Several traits are likely to become particularly relevant in the future for nGM plants, namely herbicide resistance (HR), resistance to different plant pathogens as well as modified composition, morphology, fitness (e.g., increased resistance to cold/frost, drought, or salinity) or modified reproductive characteristics. Some traits such as resistance to certain herbicides are already known from existing GM crops and their previous assessments identified issues of concern and/or risks, such as the development of herbicide resistant weeds. Other traits in nGM plants are novel; meaning they are not present in agricultural plants currently cultivated with a history of safe use, and their underlying physiological mechanisms are not yet sufficiently elucidated. Characteristics of some genome editing applications, e.g., the small extent of genomic sequence change and their higher targeting efficiency, i.e., precision, cannot be considered an indication of safety *per se*, especially in relation to novel traits created by such modifications. All nGMs considered here can result in unintended changes of different types and frequencies. However, the rapid development of nGM plants can compromise the detection and elimination of unintended effects. Thus, a case-specific premarket risk assessment should be conducted for nGM plants, including an appropriate molecular characterization to identify unintended changes and/or confirm the absence of unwanted transgenic sequences.

## Introduction

A wide range of new genetic modification techniques (nGM), which are also collectively referred to as “new techniques” or NTs in short, has been developed to modify plants for research purposes or for the development of crops (Lusser et al., 2012; Vogel, 2016; SAM, 2017). nGMs and genome editing in particular are different from conventional breeding methods and from classic genetic engineering technology and are used to produce plants with traits or a combination of traits suitable for agricultural use (Songstad et al., 2017). In recent years a number of different genome editing approaches were developed to introduce either random or directed genetic changes at specific genomic locations, particularly methods based on site-directed nucleases, e.g., CRISPR-based systems (Puchta and Fauser, 2014; Voytas and Gao, 2014; Weeks et al., 2016; Zhang et al., 2017a). Genome editing, especially approaches based on CRISPR/Cas-technology, rapidly gained prominence due to their versatility, simplicity, speed, and typically low costs. Other nGM approaches which were used to develop crop plants comprise cisgenesis, transgrafting, and approaches to support and accelerate crossbreeding schemes, such as accelerated breeding, haploid induction or reverse breeding. The latter involve genetic modification (GM) technology in intermediate steps resulting in final products that are non-transgenic, i.e., they no longer contain the inserted transgenes (Ricroch and Hénard-Damave, 2016; Schaart et al., 2016; SAM, 2017). Another motivation for plant breeders to apply such methods is that some of them, including certain types of genome editing, are not or may not be covered by biosafety legislation in certain countries (Wolt et al., 2016; Eckerstorfer et al., 2019).

Currently only limited biosafety information is available for most of the plants developed with different nGMs from risk assessment conducted for these applications. The question of whether the agricultural use of nGM plants might pose risks for the environment and/or human and animal health is mostly based on available experience with plants obtained by classic mutagenesis (particular in relation to applications of genome editing) and with transgenic plants developed by standard GM technology (e.g., in relation to cisgenesis, transgrafting, and genome editing applications aimed to integrate recombinant DNA constructs at certain genomic locations). However, the traits and unintended changes in nGM applications may differ significantly from modifications present in existing conventional or transgenic plants. Therefore, the available experience and knowledge may only be of limited value for the assessment of novel nGM plants. The availability or lack of robust biosafety information for certain nGM plants is a significant issue in the ongoing discussion concerning the regulation of nGM applications by existing biosafety frameworks, initially introduced for products developed by GM technology (Jones, 2015a; Sprink et al., 2016; Wolt, 2017) or by other legislation applicable to nGM plants used for agricultural purposes (Eckerstorfer et al., 2019).

## Overview on nGMs Covered in This Study and on Their Characteristics

Due to new developments, the spectrum of nGMs and variations thereof are increasing at a high speed (EPRS, 2016). The nGMs addressed in this study were selected based on an early and a more recent EU-level report on nGMs (Lusser et al., 2012; SAM, 2017). The following nGMs were addressed in this (see also Table 1):

- Genome editing with site-directed nucleases (SDNs), e.g., using clustered regularly interspaced short palindromic repeat (CRISPR)-directed nucleases, Transcription activator-like effector nucleases (TALENs), zinc-finger-directed nucleases (ZFNs), or meganucleases. Such SDN-based techniques can also be applied for multiplex genome editing. Other approaches were developed for base editing as well as for modification of transcriptional regulation.
- Genome editing directed by synthetic oligonucleotides, also referred to as oligonucleotide-directed mutagenesis (ODM)
- RNA dependent DNA methylation, an approach for modifying epigenetic regulation of gene expression
- Cisgenesis and intragenesis
- Transgrafting, in particular the use of GM-rootstocks in grafting
- Agro-infiltration
- Haploid induction and reverse breeding, i.e., examples of techniques developed to assist complex breeding schemes.

**Table 1:** Overview of the nGMs addressed in this study and strategy employed for literature search.

**Type of nGM**	**nGM**	**Search terms used for literature searches**
Genome editing with site-directed nucleases (SDN)	CRISPR-based systems for genome editing (CRISPR)	(crispr OR cpf1) AND (plant OR plants OR plant^*^ OR “plant breeding” OR crop^*^ OR tree^*^); (crispr OR cpf1) AND tree^*^ NOT (plant OR plants OR plant^*^ OR “plant breeding” OR crop^*^)
	TALE-directed Nuclease systems for genome editing (TALEN)	(“transcription activated-like nuclease^*^” OR TALEN OR “transcription activator-like effector nuclease^*^”) AND (plant^*^ OR crop^*^)
	Zinc-Finger-directed Nuclease systems for genome editing (ZFN)	(“zinc finger nuclease” OR ZFN) AND (plant^*^ OR crop^*^)
Genome editing directed by oligonucleotides	Oligonucleotide-directed Mutagenesis (ODM)	(oligonucleotid^*^ OR “oligonucleotide directed mutagenesis” OR ODM OR “chimeric oligonucleotid^*^” OR “chimeric RNA/DNA oligonucleotid^*^” OR chimeraplasty OR “site-directed mutagenesis” OR “gene targeting”) AND (plant^*^ OR crop^*^)
	Multiplex Automated Genomic Engineering (MAGE)	“multiplex automated genomic engineering”
Modification of gene expression	RNA-directed DNA Methylation (RdDM)	(TGS) AND (plant^*^ OR crop^*^); (RDDM OR RNA^*^directed DNA methylation) AND (plant^*^ OR crop^*^)
Variants of GM technology	Cisgenesis (CG) / Intragenesis (IG)	(cisgen^*^ OR intragen^*^ OR “all native DNA transformation” OR “all-native DNA transformation”) AND (plant^*^ OR crop^*^)
	Transgrafting (TG)	(graft^*^ AND (transg^*^ OR transform^*^ OR GM graft OR GM scion) AND (plant^*^ OR crop^*^ OR tree^*^); transgrafting applications involving GM rootstocks: (graft^*^ OR transgraft^*^ OR trans-graft^*^) AND (“GM rootstock^*^” OR “transgen^*^ rootstock”)
	Agro-infiltration (AI)	(agroinfiltr^*^ OR agroinocul^*^ OR agroinfect^*^) AND (plant^*^ OR crop^*^)
Breeding support techniques	Haploid Induction (HI)	(CENH3 OR “haploid induction” OR “genome elimination” OR haploids) AND (plant^*^ OR crop^*^)
	Reverse Breeding (RB)	(“reverse breeding” OR “crossover control”) AND (plant^*^ OR crop^*^) AND (plant^*^ OR crop^*^)

Thus, very different approaches are used to introduce genetic and phenotypic variation in plants for the development of traits of agricultural interest (van de Wiel et al., 2017). As discussed in more detail below the modifications introduced by these nGMs vary significantly from each other. We also note that these nGMs or rather the resulting plant products differ significantly from each other regarding their applicability in agriculture, as well as the associated safety issues.

Genome editing, cisgenesis, and intragenesis have in common that they introduce genetic modifications which are meant to be present in the final plant products and passed on to offspring during sexual reproduction (Holme et al., 2013). As regards genome editing a variety of different approaches are employed to achieve different types of desired modifications (Tycko et al., 2017). Approaches using SDNs and ODM are applied to introduce random (SDN-1) or directed sequence changes (SDN-2 and ODM) at specific, predefined genomic loci (Podevin et al., 2013; Sauer et al., 2016a). These approaches do not necessarily require the stable introduction of recombinant constructs into the plant genome. ODM for example is directed by small-sized synthetic oligonucleotides, which are transiently introduced into the recipient plant cells and supposed to be degraded by the cellular metabolism (Sauer et al., 2016a). SDNs which facilitate genome editing can either be inserted into the genome of the target cell as a transgene, or introduced into target cells as functional (ribonucleo-) proteins (Kanchiswamy, 2016) or expressed from transiently introduced DNA constructs (Butler et al., 2016). Some approaches for genome editing, commonly referred to as SDN-3, facilitate the insertion of transgenic constructs at specific genomic locations (Petolino and Kumar, 2016). The respective transgenic insertions are present in the final breeding product (plant or plant product) and are heritable.

Besides these basic types of genome editing a number of additional approaches, e.g., for base editing, were developed recently. Base editing uses modified SDNs, typically CRISPR variants, to modify certain DNA bases in a deliberate way (C to T or A to G) (Matsoukas, 2018; Rees and Liu, 2018).

nGMs like agro-infiltration (Vaghchhipawala et al., 2011) and transgrafting (Schaart and Visser, 2009) are typically used to modify somatic tissues or to produce chimeric plants, e.g., GM rootstocks fused to non-GM scions by transgrafting (SAM, 2017). Typically the genetic modifications introduced by these approaches are not passed on by sexual reproduction. However, the whole plant may be affected, i.e. in the above mentioned case effector substances produced in the GM rootstock may reach the upper non-GM scion and influence its phenotype (Stegemann and Bock, 2009).

RNA-directed DNA methylation (RdDM) is used to modify the expression of endogenous genes not by changing its DNA sequence, but rather through introducing epigenetic modifications which may be passed on for some generations (Mahfouz, 2010).

nGMs like haploid induction (Ravi and Chan, 2010; Britt and Kuppu, 2016) or reverse breeding (Dirks et al., 2009; Wijnker et al., 2012) are predominantly used to enable and/or speed up specific breeding schemes. They involve transgenic insertions intended to be present only at intermediate steps. Therefore, the respective transgenic modifications must be verifiably absent from the final breeding products (SAM, 2017).

## Literature Survey to Identify Applications of nGMs With Relevance for Risk Assessment

To identify nGM applications which may be relevant from a risk assessment point of view the following approach was used: different sources were screened for research on and development of plants developed by nGMs, hereinafter referred to as nGM plants, for potential future use in agriculture.

The sources included previously published reports addressing the nGMs in question, which contain information on relevant nGM plants as well as their state of development (Vogel, 2016; Hilscher et al., 2017b). Also scientific reviews addressing the use of genome editing or other nGM approaches for the development of crops for agriculture were screened for relevant information (Khatodia et al., 2016; Paul III and Qi, 2016; Hilscher et al., 2017a; van de Wiel et al., 2017). In addition the recent scientific literature was screened to identify publications addressing the use of nGMs, that were not already included in previous reviews.

The general timeframe for the literature search covered the period from January 2011 until June 2017. The searches addressing genome editing by CRISPR-based methods were limited to the period from January 2016 until June 2017, with a view to the availability of reviews covering previous years (e.g., Hilscher et al., 2017b). Relevant scientific publications from peer reviewed journals were retrieved using the databases Scopus, ProQuest Natural Science Collection, the Web of Science, and PubMed. Searches were conducted with a set of keywords relating to the individual nGMs, combined with search terms or filters to exclude applications other than plant biotechnology (see Table 1). The titles and abstracts of the references were manually screened for relevance.

The objective of the literature searches was to establish a non-exhaustive overview on recent usage of the respective nGMs. The search was not intended to establish a comprehensive collection of the whole scientific literature on nGM applications, but rather to identify the focus of current nGM approaches, the modified plant species and the developed traits. Systematic reviews of the available literature might be helpful for a more detailed discussion of specific techniques. Such a systematic review is underway for applications of genome editing in plant breeding (Modrzejewski et al., 2018). We consider that our results nevertheless broadly illustrate the significance of the different approaches in plant breeding. Our literature search also covered publications of nGM applications that are near commercialization or already commercialized in some countries, such as a herbicide-resistant oilseed rape variety developed by ODM (Gocal et al., 2015). As seen in Sovova et al. (2017) information on patents did not add significant information in terms of application and the potential for commercialization. We are thus confident that the sources we have considered sufficiently serve the purpose of this work.

In total 172 research publications addressing work in plants with all listed nGM were retrieved for the period January 2016–June 2017 (Table 2). Most of them reported the application of genome editing in different species, among them model species for research (such as *Arabidopsis* and tobacco), as well as different crop and tree species. The majority of publications (114) applied CRISPR-based approaches for genome editing. A significant focus was on the further development and adaption of CRISPR-based methods for different plant species (72).

**Table 2:** Research publications between 2011 and 2017 covering several applications for different nGMs.

** 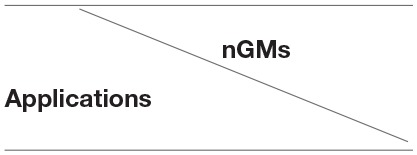 **	**Genome editing**					**RdDM**	**CG**	**IG**	TG^*^	**nGMs to support breeding**	
	CRISPR^*^	**TALEN**	**ZFN**	**MN**	**ODM**					**AI**	**HI**
**JAN. 2011–DEC. 2015**											
**Total number**	**n.a**.	**10**	**17**	**5**	**1**	**6**	**7**	**4**	**n.a**.	**14**	**9**
JAN. 2016–JUNE 2017^*^											
**Total number (172)**	**114**	**8**	**7**	**1**	**1**	**1**	**2**	**4**	**23**	**4**	**7**
SDN−1	99	5	4	−	n.a.	n.a.	n.a.	n.a.	n.a.	n.a.	n.a.
SDN−2	5	−	−	−	n.a.	n.a.	n.a.	n.a.	n.a.	n.a.	n.a.
SDN−3	4	3	3	1	n.a.	n.a.	n.a.	n.a.	n.a.	n.a.	n.a.
Base editing	4	−	−	−	n.a.	n.a.	n.a.	n.a.	n.a.	n.a.	n.a.
Other types of genome editing	2	−	−	−	n.a.	n.a.	n.a.	n.a.	n.a.	n.a.	n.a.
**OBJECTIVE OF APPLICATIONS (JAN. 2016–JUNE 2017)**											
Method development	72	1	2	1	−	−	1	1	6	−	3
Basic research	22	1	2	−	−	−	−	−	7	4	1
Applied development	20	6	3	−	1	1	1	3	10	−	2

*SDN, site-directed nuclease; CRISPR, CRISPR (Clustered regularly interspaced short palindromic repeat)-directed nuclease; TALEN, Transcription activator-like effector nuclease; ZFN, Zinc-Finger-directed nuclease; MN, Meganucleases; ODM, Oligonucleotide-directed mutagenesis; RdDM, RNA dependent DNA methylation; CG, Cisgenesis; IG, Intragenesis; TG, Transgrafting; AI, Agro-infiltration; HI, Haploid induction; Other types of genome editing: different variants of CRISPR-based genome editing, including use of nickases; n.a.: not applicable*.

* *For the use of CRISPR-based systems for genome editing and transgrafting literature was only screened for the time period Jan. 2016-June 2017*.

*Bold values indicate total numbers of publications for individual nGMs for the indicated time periods*.

This supports prior findings that CRISPR-based genome editing quickly established itself as the most important tool in genome editing (Hilscher et al., 2017a). Further variants of CRISPR-technology are continuously being developed. A small number of publications addressed the use of emerging variants of CRISPR-based systems, e.g., the use of modified or alternative CRISPR-type nucleases like Cpf1 (4) (Kim et al., 2017; Tang et al., 2017; Xu et al., 2017; Yin et al., 2017), as well as the use of modified Cas9 nucleases, e.g., as single strand nickases (2) (Schiml et al., 2014; Mikami et al., 2016) or for targeted base-editing (4) (e.g., Shimatani et al., 2017; Zong et al., 2017). This underlines the interest in the development of variants of CRISPR-based systems with increased specificity of targeting or approaches for introducing specific types of mutations at specific genomic locations, e.g., via chemical modification of specific nucleotides present in the targeted genomic sequences (Komor et al., 2016, 2017; Schaeffer and Nakata, 2016; Arora and Narula, 2017).

Significantly fewer publications addressed applications of other SDN-based genome editing methods, involving TALENs (10), ZFNs (17), and meganucleases (5). Only a single publication could be retrieved for the application of ODM between January 2016 and June 2017 (Sauer et al., 2016b). However, according to other sources these methods are actively used for the development of modified crop plants for (future) agricultural use, e.g., by companies like KeyGene and CIBUS (Abbott, 2015) as well as Calyxt in case of TALEN (Gelinsky, 2017).

SDN-1 applications clearly dominated the field of genome editing applications employing SDNs (108/130); they are applied to modify (mostly to knockout) all alleles of specific genes present in a plant line. Only 16 publications described the use of SDN-2 and SDN-3 applications; TALEN- and ZFN-based genome editing was more frequently used for SDN-3 applications (3/8 and 3/7) in comparison with CRISPR-based systems (4/114). Some of these publications describe approaches for integration and stacking of transgenes at specific, pre-modified genomic locations (“trait landing pads”) by commercial developers (Ainley et al., 2013; Kumar et al., 2015, 2016). Also the relative number of publications addressing the development of traits for agricultural use was higher between January 2016 and June 2017 for TALEN (6/8) and ZFN (3/7) when compared to the respective CRISPR applications (20/114).

When compared to genome editing (131), other nGMs were covered significantly less often in papers published between January 2016 and June 2017 (41), with transgrafting being the most prominent technique in this group (23). In that period only a few publications described approaches based on RdDM, cisgenesis, and intragenesis. However, more work using these technologies with relevance for the development of agricultural plants was published between 2011 and 2017. Publications on agro-infiltration during that period focused on its use for basic research.

The numbers in Table 2 for publications on TALEN and ZFN before 2016 correspond to the ones reported by Hilscher et al. (2017a); from January 2016 onwards a low but continuous interest remained in TALEN- and ZFN-approaches (8 and 7, respectively). Meganuclease-based systems were used less often (5 publications by the end of 2015, 1 in the subsequent period) due to the technical challenges to target different genomic sequences with this method.

The analysed literature on nGM applications in plants demonstrates that an extremely wide range of species was used in relevant research and development projects: The range includes model species for research (like *Arabidopsis* and tobacco), most crop species including important crops such as maize, rice, wheat and other cereals, soybean, potato and other plants for oilseed production as well as a broad range of vegetable and spice plants and perennial plants including fruit trees and forest trees as well as lower plants, e.g., moss species.

## Risk Assessment Considerations

### General Approach of Risk Assessment

An important aspect in the overall discussion on nGMs is whether specific biosafety issues may be associated with their plant products. To address this question two main issues have to be determined: (a) whether plant development with a particular nGM approach can lead to unintended genetic or epigenetic changes and whether they may be associated with adverse effects on human and animal health as well as the environment; (b) whether the intended use of the nGM plants may result in adverse effects related to the newly developed traits (Mahfouz et al., 2016; Bujnicki, 2017).

### Considerations Regarding Unintended Effects Associated With nGM Applications

As with GM technology or other biotechnological methods, the presently available nGMs are not sufficiently specific to introduce only the intended molecular changes into plants. Thus, a range of unintended molecular changes may be introduced by a particular nGM method and these molecular changes may lead to phenotypic effects affecting the properties of the modified plant (SAM, 2017).

In general several types of unintended effects can be distinguished (Agapito-Tenfen et al., 2018):

- Unintended changes at genomic locations other than the genomic target site(s) for intended modifications; i.e., modifications which are usually not genetically linked to the desired trait(s)
- Unintended molecular changes in the vicinity of the intended site of modification; i.e., changes different from the intended modifications, but tightly linked to the desired trait(s)
- Unintended effects different to the desired trait(s) which are due to the modifications at the genomic target; i.e., pleiotropic effects of the intended modification(s) linked to the desired trait(s).

Unintended changes may modify the expression of endogenous genes and impact the plant's metabolism and phenotype. According to the nature of the particular phenotypic effects, these unintended changes may be considered either harmless or adverse in terms of human health and the environment.

Method-related unintended molecular changes may be associated with different aspects of the overall development process of nGM products. They depend either on the mechanisms of the particular nGM or on the characteristics of further methods required for the overall development of a particular nGM plant, such as methods for *in vitro* cultivation of plant cells and tissues, methods to facilitate the uptake of nGM components (e.g., protoplast transfection methods)or methods for the regeneration of plants from cultivated cells or tissues.

Typically exogenous effector molecules need to be introduced into recipient plant cells to initiate nGM processes, such as (i) recombinant DNA constructs for stable genetic transformation of plant cells, e.g., to express nucleases for genome editing or other molecular tools required for a particular nGM; (ii) recombinant DNA constructs for transient expression of nGM-related components (RNA or proteins required for the respective nGMs); (iii) specific DNA, RNA or ribonucleoprotein complexes. Unintended genetic or epigenetic changes can be introduced as a side effect of transformation or the transfer of method-related components into the recipient cells (Latham et al., 2006; Mehrotra and Goyal, 2012).

Unintended changes may also result from the integration of genetic constructs into the recipient genome of plant cells for nGM approaches that involve the use of GM techniques. This relates to e.g., cisgenesis/intragenesis, the transformation of rootstocks for transgrafting and genome editing approaches that are based on the expression of SDN components from transgenic constructs. It is typically a random process and thus can result in unintended genetic changes, e.g., by the disruption of functionally important genomic sequences or due to the integration of other unrelated DNA sequences (SAM, 2017). Untargeted integration of non-endogenous sequences can also modify the expression of endogenous genes located in the vicinity of the integration site(s) (Ladics et al., 2015).

It should also be noted that genetic constructs that are only transiently introduced into plant cells to express method-related components may integrate into the genome of the recipient cells. If transgenic constructs should only be present during intermediate steps it is important to assess whether all such modifications are indeed fully removed and absent from the final product. This relates to any inserts of the constructs for expression of method components as well as to secondary inserts, e.g., of vector backbone sequences. Braatz et al. (2017) for example found by way of whole-genome sequencing that transformation of oilseed rape with an CRISPR-Cas9 expression construct resulted in at least five independent insertions of vector backbone sequences in the genome of the modified plant.

Unintended genetic and epigenetic changes may also result from the respective particular nGM mechanism. Well-known examples are off-target modifications associated with approaches for genome editing. They typically happen in genomic sequences that share a sufficiently high degree of similarity with the target loci and thus can associate with the molecular editing tools leading to off-target edits (Kanchiswamy et al., 2016; Yee, 2016). Off-target activity can also be associated with other nGM, e.g., RdDM approaches. In such cases not only the target site(s) are epigenetically modified, but also other genomic loci (Galonska et al., 2018).

The frequency of off-target effects as well as their extent and distribution in the genome are different for the various genome editing approaches and depend on both the targeting characteristics of the particular approach and on the specific method used for genome editing (HCB, 2017; Wolt, 2017), including the exact experimental protocol (Yee, 2016).

From a risk assessment point of view it is relevant to assess whether the respective unintended molecular changes are leading to phenotypic changes of an adverse nature (SAM, 2017). Off-target modifications, which result in readily detectable phenotypic changes, can be identified and possibly eliminated during downstream breeding when generating elite lines (Zhao and Wolt, 2017). Significant alterations of important agronomic parameters, such as yield, fitness, growth, and reproduction may be detected quite readily. However, not all induced phenotypic changes can be easily detected. Subtle changes e.g., in composition are more difficult to detect, however they may impact the nutritional quality or may be associated with allergenic or toxic effects. Also, some unintended changes may be genetically tightly linked to the desired trait(s) while others are not. That does influence how easily they can be removed, if at all. The probability that unintended changes are indeed removed depends critically on the number of breeding steps involved to establish a final breeding product. While this is less of a concern with annual crop plants which are typically subjected to a sufficient number of breeding cycles, this constraint is relevant for plants like trees, which do not undergo the same number of breeding cycles for practical reasons, as well as for plants which are mostly propagated vegetatively. On the other hand nGMs like genome editing may be used for direct modification of elite lines to speed up breeding processes, according to information presented at a recent conference (OECD, 2018). However, faster ways of plant breeding may negatively impact the ability to safely remove any unwanted unintended modifications. Thus, strategies to minimize off-target activity and to identify unintended modifications should be implemented for the use of genome editing approaches to produce modified plants (SAM, 2017).

Most nGM approaches require the use of further techniques to cultivate cells or explanted tissues (embryogenic or somatic tissues used for callus transformation or plant cells treated to yield protoplasts to facilitate transfection of genetic material or other method-related components), and methods to regenerate modified plants from single cells. A fair number of the genome edited plants reported in Bortesi and Fischer (2015) as well as Schaeffer and Nakata (2016) involved protoplast transfection which was used to deliver the genetic constructs for the expression of SDN-reagents. Plant protoplast technology is also involved in DNA-free methods for genome editing. For such approaches functional site-directed nucleases, mostly CRISPR-ribonucleoproteins, are introduced into protoplasts to initiate editing (Malnoy et al., 2016; Kim et al., 2017). These approaches are currently considered and promoted as alternative to genome editing applications involving the delivery of DNA (Kanchiswamy, 2016; Ran et al., 2017). However, it is known that techniques such as protoplast technology, *in vitro* cultivation of cells and regeneration of plants from cells and tissues are associated with unintended genetic changes (Filipecki and Malepszy, 2006; Bairu et al., 2011; Ladics et al., 2015; HCB, 2017). These techniques can induce somaclonal variation which adds to the range of random genetic changes introduced by nGMs. While somaclonal variation is not a specific feature of nGM approaches, but can also happen in conventional breeding involving cell and tissue cultivation steps, some nGM methods dependent on methods known to promote somaclonal variation. It should thus be ensured that such changes are eliminated during subsequent steps of the breeding process.

Some types of genetic modification can also give rise to pleiotropic effects, i.e., unintended secondary phenotypes which are also determined by the modified gene(s) and which are expressed along with the desired trait (SAM, 2017). Pleiotropic effects can occur with traits developed by all types of breeding approaches, including nGMs. Pleiotropic effects will be present in the final breeding products, since they are tied to the desired trait(s). An example are nGM plants which were modified for increased disease resistance due to the inactivation of susceptibility genes, namely the *mlo* genes conferring broad-spectrum resistance against powdery mildew fungi (Kusch and Panstruga, 2017). A range of pleiotropic effects was found to be associated with the inactivation of certain *mlo* genes, including yield decrease and increased susceptibility to other fungal pathogens as well as effects on mycorrhizal development in roots (Brown and Rant, 2013). Data gathered in the course of screening for unexpected effects during the development process of nGM plants can support the risk assessment of unintended pleiotropic effects conducted in accordance with guidance established by EFSA (EFSA, 2010).

Unintended effects may also be based on modifications/alterations, in particular disruption, of endogenous genomic sequences in proximity to integration sites of DNA introduced to develop plants by certain nGMs. Applications of cisgenesis, intragenesis, or SDN-3 applications may be associated with such effects, depending on the characteristics of the integration site. Due to the genomic proximity of the integrated genetic elements and the altered genomic sequences flanking these elements, such unintended modifications cannot be removed by segregation during further breeding steps. Provided that their functions are understood the molecular characterization of the genomic sequences altered during the integration can provide indications as to whether unintended effects may arise. It may even be possible to predict the phenotype that may result from the modification.

For the purpose of a comprehensive risk assessment of nGM plants unintended effects associated with all technical interventions involved in the process to develop a specific nGM plant have to be considered. A particular focus should be on unintended effects that may be predicted based on the specific characteristics of certain nGMs, such as off-target effects associated with a particular approach for genome editing. This can be addressed through an appropriate molecular characterization of the nGM application taking into account all procedures that were used to establish the nGM application in question. Information from the molecular characterization can then be used to address the question of whether the identified molecular changes may be tied to potential effects at the phenotypic level that should be further assessed.

### Considerations Regarding Traits Developed by nGMs

For a comprehensive assessment the risks associated with the newly developed nGM plants and their use in particular (agricultural) environments need to be considered. The new traits generated by an nGM can influence the species-specific characteristics of modified plants and are thus highly important for the assessment of overall risks.

nGMs and genome editing in particular can be used to introduce traits already present in wild populations or related species in a fast and straightforward way. Some of these traits may alternatively be introduced with either conventional breeding or GM technology, though, nGMs in many cases have technical advantages, e.g., providing a simpler, faster, and less costly approach (HCB, 2017). However, many of these traits developed with genome editing and other nGMs need to be considered novel concerning their use in crop plants. Such traits are not present in stable, cultivated populations of the plant species at significant levels (HCB, 2017). For plants with such novel traits typically only limited knowledge and experience concerning their (environmental) effects are available and no history of safe use. In regulatory frameworks which are based on novelty as a product-oriented regulatory trigger, i.e., in Canada, this aspect is crucial for the denomination of products which are subject to oversight for biosafety, e.g., according to the “Plants with novel traits (PNT)”-Regulations (Shearer, 2014). Canada also regulates PNTs which are generated by conventional plant breeding approaches for biosafety (Eckerstorfer et al., 2019).

The available literature on nGM plants provides indications, regarding what traits are currently developed with different nGMs. For a discussion of the associated risks they are grouped into the following classes:

1. Herbicide resistance (HR)
2. Disease resistance (to viral, bacterial, and fungal plant pathogens)
3. Altered composition
4. Enhanced fitness against environmental stressors and alteration of morphological or reproductive plant characteristics.

In the following sections examples of nGM plants for each trait class are presented. Where available, applications with an advanced stage of development are included. The examples are not meant to be exhaustive, but rather to highlight that a case-specific assessment of applications of the respective class is warranted. A significant number of these traits are developed using different types of genome editing. However, for technical, legal or other reasons also other nGM approaches are used to generate plants with traits from all of the four classes.

Safety considerations associated with applications of genome editing or other nGMs, like transgrafting or cisgenesis/intragenesis, should be based on the characteristics of the particular application. Due to their different modes of action the particular issues for risk assessment can be very different.

#### nGM Plants With Herbicide Resistance

nGMs are used to develop resistance to a number of different herbicides in several agricultural crops:

- Resistance to acetolactate synthase (ALS) inhibiting herbicides was established via ODM in oilseed rape (Gocal et al., 2015), by SDN-1 technology in potato with TALEN (Nicolia et al., 2015; Butler et al., 2016) and CRISPR/Cas9 (Butler et al., 2016), in rice with TALEN (Li et al., 2016a) and in tobacco with ZNF (Townsend et al., 2009). In Chinese cabbage this trait was introduced by cisgenesis (Konagaya et al., 2013).
- Resistance to glyphosate-based herbicides was developed by intragenesis in strawberries (Carvalho and Folta, 2017) and in flax by ODM (Abbott, 2015), as well as by a combination of ODM and CRISPR/Cas9 (Sauer et al., 2016b).
- Furthermore genome editing approaches based on SDN-2 and SDN-3 technology were developed for the targeted introduction of (multiple) herbicide resistance genes: resistance to glufosinate ammonium and 2,4 D herbicides in maize with ZFN (Ainley et al., 2013), resistance to glyphosate-based herbicides in cassava using CRISPR/Cas9 (Chauhan et al., 2017), in cotton with meganucleases (D'Halluin et al., 2013) and in maize using ZFN (Kumar et al., 2016). Resistance to bialaphos was developed in tobacco using ZFN (Schneider et al., 2016). Resistance to ALS-inhibiting herbicides via SDN-2 CRISPR/Cas9 technology was developed in maize (Svitashev et al., 2015, 2016) as well as via SDN-3 in rice (Sun et al., 2016) and soybean (Li et al., 2015).

Experience with effects resulting from these traits is available from existing risk assessments of herbicide resistant GM plants. Of particular relevance are indirect effects on biodiversity resulting from the changes in weed management, and the development of herbicide resistant weeds (EFSA, 2010; Schütte et al., 2017). For herbicide resistant oilseed rape, experience is available from comparable conventional HR crops, indicating a number of concerns, e.g., dispersal and persistence of HR volunteers (Expertgroup, 2014; Huang et al., 2016). As noted by Ishii and Araki (2017) ALS-resistant rice which was cultivated in Italy and the USA hybridized with related wild species and HR resistant weeds emerged from these outcrossing events. This underlines the fact that the assessment of the herbicide resistance trait is important independent of the method or technology that was used to produce the crops.

It has been shown recently in *Arabidopsis* that elevated expression levels of modified EPSPS can lead to pleiotropic effects, like elevated auxin content and increased fecundity of the modified plants (Fang et al., 2018). To ensure food and feed safety the absence of unintended effects on composition should be confirmed for respective HR nGM crops.

Some GM crops, in particular soybean, have been made resistant to multiple herbicides, including glyphosate, glufosinate ammonium, dicamba and others (see e.g., http://bch.cbd.int/database/lmo-registry/). Such crops can be expected to contain cocktail mixes of pesticide residues. After methods to assess the cumulative and synergistic effects of pesticides were developed (EFSA, 2013), they have to be taken into account for risk assessment and potential human health impacts (Regulations (EC) No. 396/2005 and No. 1107/2009). A report by the European Food Safety Authority (EFSA) on how to consider effects of pesticide cocktails on the nervous system is about to be finalized (see information at: http://www.efsa.europa.eu/en/consultations/call/180508-0). As mentioned above, maize with two HR genes was already developed using ZFN. Therefore, cocktail mixes of pesticide residues can be expected to become a relevant risk assessment issue for nGM crops as well.

The herbicide resistant oilseed rape from Cibus developed using ODM is the only HR-nGM plant which is actually cultivated so far, but other crops with similar traits are in the commercial pipeline. It can be expected that herbicide resistant nGM crops will continue to be an important objective for future commercial plant development (Kaskey, 2018).

#### nGM Plants With Disease Resistance

A number of different approaches were developed for increased resistance of plants against different viral, bacterial and fungal pathogens. Approaches included

- Knockout by genome editing approaches of plant susceptibility factors for bacterial and fungal pathogens in grapefruit, wheat, tomato, grapevine, apple, and rice (Wang et al., 2014, 2016; Jia et al., 2016, 2017; Malnoy et al., 2016; Blanvillain-Baufum et al., 2017; Nekrasov et al., 2017; Zhang et al., 2017b) or knockout of viral host factors in *Arabidopsis* (Pyott et al., 2016) and cucumber (Chandrasekaran et al., 2016). Most of these applications were developed with SDN-1 approaches using CRISPR-based methods (Jia et al., 2016, 2017; Wang et al., 2016; Zhang et al., 2017b) or TALENs (Wang et al., 2014; Blanvillain-Baufum et al., 2017).
- Expression of resistance genes in apple, potato, and grapevine (Vanblaere et al., 2011, 2014; Haverkort et al., 2016) and antimicrobial substances against fungal pathogens (Rubio et al., 2015) by transgrafting and cisgenesis applications.

Resistance to powdery mildew, a fungal disease, was established by knocking out plant susceptibility genes by genome editing. However, a number of pleiotropic effects such as reduced plant size or premature senescence were described (Kusch and Panstruga, 2017) most likely because the knocked out plant genes may have several other functions as well. Also knockout or silencing of members of the *mlo* gene family that are not involved in pathogen susceptibility by off-target activity may lead to unintended effects on physiology, development or composition with implications for food, feed and environmental safety (Pessina, 2016).

Other aspects have to be considered for applications to induce virus resistance by transgrafting. Lemgo et al. (2013) identified several concerns, that should be addressed during risk assessment: These include pleiotropic silencing effects, effects of the transgenic rootstock on non-target organisms, e.g., on soil organisms, gene transfer of virus resistance to wild type plants resulting in increased fitness and invasiveness, potential development of novel viral strains and food safety effects. For transgrafting applications in general the potential mobility of the transgenic product across graft junctions influences the likelihood for environmental or food safety risks (Schaart and Visser, 2009; Song et al., 2015).

#### nGM Plants With Compositional Changes

A variety of nGM plants with changed composition were developed mostly by genome editing approaches and some by cisgenesis/intragenesis. Examples of targeted traits were among others:

- CRISPR/Cas-mediated (SDN-1) changes of sugar and starch content in potato and rice (Andersson et al., 2017; Sun et al., 2017).
- CRISPR/Cas-mediated SDN-1 knockouts of genes resulting in altered lipid composition, e.g., in *Camelina* (Jiang et al., 2017; Morineau et al., 2017) and soybean (Haun et al., 2014; Demorest et al., 2016; Kim et al., 2017).
- CRISPR/Cas-mediated SDN-1 genome editing to reduce browning in mushrooms (Waltz, 2016), TALEN-mediated reduction of lignin in sugarcane (Jung and Altpeter, 2016), ZFN-based reduction of phytate in maize (Shukla et al., 2009) and TALEN-mediated reduction of components which reduce the storage capacity and processing quality of potatoes (Clasen et al., 2016) and rice grain (Ma et al., 2015).
- CRISPR/Cas-mediated silencing of several different α-gliadins in wheat, resulting in a reduction in the content of anti-nutritional gluten (Sanchez-Leon et al., 2018).
- TALEN-mediated SDN-1 knockout of genes modifying the content of substances increasing fragrance in rice (Shan et al., 2015).
- Cisgenic modification to increase the anthocyanin content in apple (Schaart et al., 2016) and to reduce the acrylamide-forming potential of potato tubers during processing (Chawla et al., 2012).

Based on experience with problem formulation for the risk assessment of GM plants (EFSA, 2011) a number of potential risk issues as regards food and feed safety and environmental effects should be addressed in the risk assessment of nGM plants from this class, particularly any toxic or allergenic effect resulting from proteins with modified sequence, or any anti-nutritive effect of newly produced compounds. Compositional changes can furthermore result in environmental effects due to altered interactions with herbivorous animals, e.g., for nGM plants with increased sugar content, or by effects on morphological characteristics, like stability, e.g., for nGM plants with reduced lignin content.

#### nGM Plants With Enhanced Fitness Against Environmental Stressors and Alteration of Morphological or Reproductive Plant Characteristics

Several approaches including genome editing applications and transgrafting were used to establish a variety of different traits with environmental/ecological relevance:

- Transgrafting in tomato (Nakamura et al., 2016) and genome editing in *Arabidopsis* to improve abiotic stress response e.g., to cold, drought, salinity (Osakabe et al., 2016; Zhao and Zhu, 2016).
- CRISPR/Cas9-mediated SDN-1 knockout of two ALCATRAZ genes for increased seed shatter resistance of oilseed rape (Braatz et al., 2017).
- CRISPR/Cas9-mediated SDN-1 knockout in tomato of a factor (SlAGL6) influencing early maturation and facultative parthenocarpy for fruit production under climate (heat) stress (Klap et al., 2017) and of a flowering repressor (SP5G) for early flowering (Soyk et al., 2017).
- CRISPR/Cas9-mediated SDN-1 type alterations of the SlCLV3 promoter in tomato to generate larger fruit and increased numbers of flower buds (Rodríguez-Leal et al., 2017).
- CRISPR/Cas9-mediated SDN-1 editing of several genes in rice to generate early-maturing cultivars (Li et al., 2017), and in genes acting as regulators of grain number, panicle architecture, grain size, and plant architecture to create mutants showing enhanced grain number, dense erect panicles, and larger grain size (Li et al., 2016b) as well as modification of three negative regulators of grain size in a multiplex approach (Xu et al., 2016).

Traits related to enhanced fitness can result in adverse effects due to an increased potential for invasiveness or weediness in the modified plants or sexually compatible species following introgression of such traits. However, depending on the modified trait and the wild relative, effects of outcrossing can be adverse for different reasons: in the case of related valued species a decrease in reproduction or fitness would be regarded as adverse, similar as an increase of reproductive fitness in case of the weedy relatives.

Two recent publications (Li et al., 2018; Zsögön et al., 2018) indicate the potential of genome editing for an approach called *de novo* domestication, i.e., to rapidly develop crop lines from wild forms with desired properties like strong resistance toward pathogens or salt tolerance. In both cases characteristics associated with domesticated tomato plants were established in different lines of *Solanum pimpinellifolium* by simultaneously editing only 4 or 6 genomic loci, respectively, while maintaining the desired resistances present in the wild lines. Among the introduced domestic characteristics were increased fruit number, size, shape and nutrient content of fruits as well as plant architecture and growth characteristics. The authors regard their approach as a viable route for the direct development of new crop varieties from wild plants in order to exploit their genetic diversity and thus as a fast and simple alternative to classic breeding programs. However, also any potential hazards associated with the agricultural use of such novel crops with wildtype genetic backgrounds need to be carefully assessed.

### nGM Characteristics Relevant for Risk Assessment Considerations

#### Combination of Biotechnological and Conventional Methods

The scientific literature considered in this study demonstrates that in most cases specific nGMs are not used in isolation, but various biotechnological methods are combined in the different breeding processes to establish nGM applications. The following examples of the combined application of different methods for the development of nGM applications illustrate the various relationships.

In many approaches GM technology is used at some point to establish intermediate or final products containing transgenic insertions. Typically such approaches are used to transfer and express the molecular tools necessary for the development of a variety of nGM applications. This includes e.g., expression of site-directed nuclease components for genome editing approaches, expression of transgenes in the modified rootstocks (or other parts) of plants established by transgrafting or during intermediate steps in the development of plants utilizing nGM approaches to speed up breeding cycles, e.g., accelerated breeding (Zhang et al., 2010). For haploid induction, reverse breeding and accelerated breeding as well as for most products developed by SDN approaches for genome editing, the recombinant components are first integrated into the genome of the plant to be modified and then removed by segregation during later steps to derive the final breeding products.

Likewise nGMs may be used as technical tools to support the application of another nGM category. For instance genome editing can be used to knockout specific endogenous plant genes, e.g., to initiate early flowering as a tool for developing products by accelerated breeding (Zhang et al., 2010), or to suppress meiotic recombination in plants which are used in reverse breeding applications (Dirks et al., 2009). CRISPR-based systems in combination with DNA methyltransferases can be utilized for targeted modification of genomic methylation patterns to change the expression of targeted genetic elements (Guha et al., 2017).

Genome editing of type SDN-3 is used to support the targeted insertion of transgenes at specific chromosomal loci and for molecular stacking of multiple transgenes (Ainley et al., 2013; Kumar et al., 2015). Such approaches may be similarly used for targeted insertion of cisgenic or intragenic constructs (AGES, 2013).

Sauer et al. (2016b) and Rivera-Torres and Kmiec (2016) point out that ODM may be simultaneously applied with SDN-techniques to make genome editing applications more efficient. Typically, the rate of sequence change by ODM is quite low, but is substantially increased, when double-strand breaks are introduced in close vicinity to the ODM target site.

Other nGMs, such as agro-infiltration (Vogel, 2012) and/or the use of viral vectors for gene transfer and expression of method related components (Butler et al., 2016; Lozano-Duran, 2016), are used as tools for transient gene expression in plant cells for two different purposes: (i) as a tool to study the effects of expression of a specific gene or genetic construct in a target crop, or (ii) as a tool to express molecules like dsRNAs or site specific nucleases which then initiate the further biotechnological modification of the respective crops, e.g., by RdDM or genome editing. Examples for (i) are e.g., the use of agro-infiltration to study the effects of transgenes involved in fatty acid metabolism (Grimberg et al., 2015), other examples are provided in Vogel (2016). Examples for (ii) are e.g., approaches for the expression of site specific nucleases as well as of donor DNA constructs required for SDN-2 and SDN-3 applications to initiate genome editing in the target plants (Baltes et al., 2014). Currently methods are developed to use viral vectors for plant modification in the environment, relying on insects to disseminate the viral vectors in the field (DARPA, 2016).

nGMs such as CENH3-mediated haploid induction (HI) were developed for the fast production of homozygous lines from a heterozygous parent without the need for lengthy back-crossing cycles. The method induces the *in vivo* production of haploid offspring from crosses between a haploid inducer line and a wildtype parent. Double-haploid plants containing two identical sets of chromosomes can then be generated from the haploid lines in a second step. Haploid induction can be used to e.g., produce homozygous plant lines from genome edited plants (Gurushidze et al., 2017). However, CENH3-mediated haploid induction could be applied as a general tool to speed up all breeding activities by substituting time-consuming back-crossing steps with the faster HI approach.

As already mentioned, conventional methods are typically used in all nGM approaches. Particular methods, e.g., *in vitro* culturing of isolated plant cells or tissues or protoplast technology, are associated with a different potential for inducing unintended modifications, especially the introduction of random genetic changes unrelated to the intended modifications (Filipecki and Malepszy, 2006).

#### Specificity of Genome Editing vs. Off-Target Effects

Any method for altering the genetic make up of plants, including conventional breeding, which is not sufficiently specific to induce only the desired genomic modifications is associated with unintended effects (Ladics et al., 2015). nGM are no exception to this rule, even if some of them, e.g., genome editing, are significantly more specific compared to other methods including GM technology and classical mutagenesis. Recent technical reviews note that different nGM approaches achieve different levels of precision, i.e., specificity of targeting (Agapito-Tenfen and Wikmark, 2015; Hilscher et al., 2017b; SAM, 2017).

Likewise the various types of genome editing are dissimilar in terms of the number of unintended effects due to off-target activity. Some factors which influence the level of off-target activity and thus the precision or rather the efficiency of the particular approach were identified (Yee, 2016; Zhao and Wolt, 2017). According to Yee (2016) off-target activity depends on

1. the frequency of homologous sequences in the genome
2. the characteristics of the specific nuclease type
3. the expression level of the nuclease
4. the time span for which the nuclease is present in the target cell
5. the accessibility of the homologous sequence and of any potential off-target sequences in the chromatin.

The accessibility of DNA genomic regions to some nucleases used in genome editing, especially to MNs, ZFNs, and TALENs, depends e.g., on their specific methylation pattern (Guha et al., 2017). Other factors influencing off-target activity are explained in the following.

In recent years CRISPR-nuclease variants with enhanced specificity were developed to reduce off-target activity, such as a modified, high-fidelity Cas9 or nucleases from other bacteria with an intrinsically higher specificity, e.g., Cpf1 (Kleinstiver et al., 2016; Zhao and Wolt, 2017). Unwanted off-target activity could be reduced through transient expression of nuclease components and by expression at reduced levels and in specific cell-types or developmental stages (Yee, 2016). Also various other methods are developed to limit the activity of SDNs in target cells, including the use of inducer or repressor molecules to control the expression or activity of the respective nucleases (Pawluk et al., 2016). Furthermore, fewer off-target changes occurred when functional nuclease molecules were preassembled and directly introduced into recipient cells, instead of delivering SDN-components as genetic constructs (Guha et al., 2017; Hilscher et al., 2017b; Liang et al., 2017).

Different approaches may be used to limit the off-target activity of SDN-mediated genome editing. First developers can select and apply suitable methods with a high level of specificity taking into account the above mentioned factors. Furthermore, off-target activity can also be influenced by the choice of the specific genomic target sequence, e.g., by selecting target sequences which display a low homology to other genomic sequences, in order to limit the number of unintended binding sites throughout the respective plant genome.

Bioinformatic tools and special software help to predict genomic target sites and design suitable SDNs described in Kanchiswamy et al. (2016) and Zhao and Wolt (2017). There are concerns, however, that such *in silico* screening/identification for off-target sites may not reliably identify all *in vivo* off-target sites. Thus, for genome editing of animal cells new approaches have been suggested (see e.g., Akcakaya et al., 2018), which may be also employed for genome editing to modify plants. In addition calls have been issued to also consider and investigate potential target sites with lower cutting probabilities (Chakraborty, 2018).

A suite of *in vitro* methods is available to identify sites of potential off-target activity in the genome; some of them, including Genome-wide, Unbiased Identification of DSBs Enabled by Sequencing (GUIDE-seq), High-Throughput Genomic Translocation Sequencing (HTGTS), Breaks Labeling, Enrichments on Streptavidin and Next-Generation Sequencing (BLESS), and Digested Genome Sequencing (Digenome-seq), can provide unbiased whole genome screens for such sites (Kanchiswamy et al., 2016; Zischewski et al., 2017). Additionally the final genome edited plants can be checked with whole genome sequencing and biochemical methods for potential off-target modifications (Zischewski et al., 2017). However, testing by whole genome sequencing may be constrained by technical limitations, e.g., if sequence information from repetitive sequences cannot be obtained (SAM, 2017). If adequate reference genomes are not available additional efforts to generate whole genome data from the parental line are required to conduct the comparison to identify unintended sequence changes.

In recent years a number of genome editing applications in plants were checked for off-target changes. Hilscher et al. (2017b) concluded that overall levels of untargeted mutational changes throughout the plant genome were not elevated. However, their review included several reports that identified off-target edits at genomic locations which were very similar to the target sequence (see Hilscher et al., 2017b). Another report noted unexpectedly high off-target activity (Zhang et al., 2016b). Furthermore, recent research has shown that assumptions regarding the level of specificity associated with a particular SDN may not always hold true. In a specific case a modified Cas9 nuclease with less stringent requirements for matching a specific protospacer adjacent motive (PAM) unexpectedly displayed a higher overall specificity (Hu et al., 2018). Recent reports from genome editing experiments in mammalian cells indicate that significant numbers of larger deletions were caused by CRISPR/Cas9-mediated genome editing using different methods, including stable transformation with SDN-expression constructs, transient expression of CRISPR/Cas and transfection with functional CRISPR-Ribonucleoprotein complexes (Kosicki et al., 2018). In addition to genetic modifications at target sequences different kinds of secondary modifications (point mutations, indels, deletions and insertions) were found at distant genomic loci (Kosicki et al., 2018). It needs to be seen whether these results are also relevant for plant systems. However, it illustrates that assumptions regarding the high degree of specificity of genome editing approaches may not hold true as a general rule. It also underlines that current knowledge concerning prediction and detection of off-target modifications associated with genome editing is still limited and needs to be improved (Wolt, 2017).

Uncertainties that remain regarding the occurrence of unintended effects cannot sufficiently be addressed by a rational design of the methods for genome editing at the time being. Rather developers still have to resort to empirical testing of the efficiency and specificity of different method variants approaches to select methods with a good ratio of on-target efficacy vs. off-target activity, e.g., as described by Kleinstiver et al. (2016). Similarly appropriate approaches for the molecular characterization of nGM plants should be implemented to identify unintended effects during risk assessment. The results can then be addressed by a targeted phenotypical assessment to determine the significance of the unintended effects identified. The existing principles for risk assessment established for GMOs provide a general framework for this. However, specific guidance for this approach is needed, but not yet available.

#### Depth of Intervention

Genome editing applications of SDN-1 type introduce small sized, random sequence changes or even point mutations at targeted genomic locations. Due to the characteristics of the changes introduced by SDN-1 applications, they were compared with plants carrying spontaneous mutations or plants produced by classical mutagenesis (Pauwels et al., 2014). However, spontaneous mutations and classical mutagenesis are neither directed nor targeted. Both widen the genetic diversity of plants in the first step and then breeders select plants with desired phenotypical modifications in a second step. As outlined below, certain SDN-1 applications, particularly applications to introduce multiple modifications at different genomic targets, can result in substantial metabolic reprogramming; this is generally overlooked when SDN-1 applications are merely judged by the small extent of genetic change introduced at single target sites.

Analysis of current developments show that several SDN-1 type applications aim to simultaneously introduce modifications (i) into multiple alleles, (ii) into all members of a gene family or (iii) into different functional genes (Khatodia et al., 2016; Paul III and Qi, 2016). This is also called multiplexing (Khatodia et al., 2016; Paul III and Qi, 2016). In particular CRISPR-based systems for genome editing provide a platform to achieve fast and efficient multiplexing in plants or other organisms (Lowder et al., 2015; Qi et al., 2016; Zhang et al., 2016b; Zetsche et al., 2017).

Proof-of-concept studies for multiplexed approaches with different site-directed nucleases were conducted in various crops, including maize (Qi et al., 2016), rice (Xu et al., 2016) and wheat (Wang et al., 2014; Gil-Humanes et al., 2017). In rice up to 21 different target genes were modified in a single step (Liang et al., 2016). In a recent study in wheat 35 different alpha-gliadin genes out of the 45 genes present in a wildtype line were knocked out using a multiplexed approach (Sanchez-Leon et al., 2018). Sanchez-Leon et al. (2018) suggest that multiplexed genome editing approaches can provide a route to develop low gluten wheat, something which has not been achieved by traditional plant breeding and mutagenesis approaches so far.

In the initial phase most genome editing applications addressed single genomic targets, i.e., single genes or all alleles of single genes. However, modifying complex polygenetic traits, like the gliadin content in wheat, requires simultaneous modification of multiple different genomic targets. For a significant number of multiplexed genome editing approaches no comparable products exist, that were developed by other approaches. Conventional approaches were used for such purposes only in few cases, such as a TILLING approach to introduce multigenic powdery mildew resistance (Acevedo-Garcia et al., 2017). Therefore, mostly no history of safe use is available for products of multiplexed applications of genome editing.

Further examples of multiplexed genome editing approaches address environmental stress response, plant development and composition:

- Knockout of transcription factors CBF1/2/3, that directly regulate cold responsive genes in *Arabidopsis* (Zhao et al., 2016; Shi et al., 2017b)
- Targeting of six of the 14 PYL ABA receptor genes in *Arabidopsis* to assess their functional importance e.g., for root elongation and plant growth (Zhang et al., 2016b)
- Knockout of two ALCATRAZ (ALC) homoeologs involved in regulation of seed shattering of mature fruits in oilseed rape (Braatz et al., 2017)
- Knockout of four closely related rice MPK genes essential for rice development (Minkenberg et al., 2017)
- Knockout of three flowering suppressor genes that negatively control the heading date of rice varieties (Li et al., 2017)
- Targeted mutagenesis of the three delta-12-desaturase (FAD2) genes to modify oil composition in *Camelina* (Jiang et al., 2017; Morineau et al., 2017)
- Targeted mutagenesis of the FAD2-1A and FAD2-1B genes to establish soybean varieties low in polyunsaturated fatty acids (Haun et al., 2014).

Chari and Church (2017) assume that the current approaches are only a first step to future large scale engineering of metabolic pathways and improved resistance to disease and environmental stress. They envision the application of extensive, but highly specific multiplexed genome editing in target organisms with the help of template DNAs, either fully synthetic or extensively remodeled by MAGE (“multiplexed automated genome engineering”) in a prior step (Wang and Church, 2011). Until now MAGE was not applied directly to plants.

The phenotypic outcomes of complex multiplexed interventions may not be fully predictable based on currently available information. In those cases further information and testing is necessary, e.g., based on the existing framework of GMO risk assessment. In addition presentations at a recent conference (OECD, 2018) indicated that the overall efficacy of multiplexed editing approaches is still quite low. Low efficacy of approaches however could compromise their specificity and the low relative frequency of unintended changes. The removal of unintended modifications through crossbreeding is more difficult to achieve for multiplexed approaches, since several different modified genes need to be retained in the final breeding product. Thus, a sufficient molecular and phenotypic characterization is required to assess the effects of the genetic modifications on physiological functions. These considerations are not specific for multiplexed genome editing, but apply likewise to all nGM approaches resulting in complex and novel types of outcomes, e.g., modifications that result in manifold changes of gene expression in the respective plants or approaches for *de novo* domestication (see nGM plants with enhanced fitness against environmental stressors and alteration of 491 morphological or reproductive plant characteristics).

### Risk Assessment for nGM Crops According to the EU Regulatory Framework

Until the recent ruling of the Court of Justice of the European Union (ECJ, 2018) considerable legal uncertainty remained concerning the regulatory status of nGM applications, genome editing in particular (Jones, 2015b). Consequently it was also unclear whether risk assessment requirements for GMOs according to Directive 2001/18/EC would apply for nGM plants or not.

The ECJ ruled that organisms obtained by mutagenesis are GMOs and in principle subject to the obligations of Directive 2001/18/EC (ECJ, 2018). The Court considered that the risks of the use of new techniques of mutagenesis might prove to be similar to those resulting from the release of GMOs developed by transgenesis. Indeed, many of the risk hypotheses e.g., considered by EFSA for GM plants (EFSA, 2010, 2011) are also relevant for nGM plants with traits directed to increase environmental fitness to abiotic stress, diseases or pests, as well as traits for changed composition and herbicide resistance. The ECJ also referred to the novelty of nGMs, i.e., their lack of a long safety record, and their potential to produce GMOs at a significantly faster rate compared with methods of conventional mutagenesis. The Court's ruling is based on a legal analysis of the current regulatory framework in the EU, i.e., Directive 2001/18/EC. It concludes that applications of genome editing should undergo a premarket risk assessment and be subject to risk management as appropriate.

The court ruling was met with quite some astonishment and policy makers were called to amend Directive 2001/18/EC to exclude genome editing applications from regulation (Purnhagen et al., 2018; Urnov et al., 2018). Preliminary proposals toward this have already been submitted by the Netherlands, but have been met with mixed enthusiasm and support. Therefore, it remains to be seen whether amending Directive 2001/18/EC will happen in the near future. From a risk assessment point of view excluding any genome editing approach from biosafety regulation right now would have significant consequences for the standard and quality of assessment which is provided for these applications: Other sectoral EU regulations which apply to all agricultural and food products, among others the EU Novel Food Regulation No. (EU) 2015/2283 or the regulatory requirements for registration of plant varieties in EU or national catalogues, fail to provide for a breadth and standard of risk assessment comparable with the requirements according to the respective biosafety frameworks (Spranger, 2017; Eckerstorfer et al., 2019).

### Toward a Case-Specific Framing of Risk Assessment

At present risk assessors and regulators face a number of challenges when considering which specific biosafety issues need to be addressed for nGM applications.

One major challenge is that the fields of nGMs in general and genome editing in particular are complex and rapidly developing. The overall range of such nGMs is very broad and is expanding rapidly. The various methodologies used for crop modification aim at different breeding objectives and thus result in products with significantly different traits and characteristics. A common risk assessment framework for all nGM plants therefore needs to take into account the range of methods used and the range of traits introduced. Not all plants developed by a particular nGM approach will be associated with a similar level of risk. Consequently potential risks of a nGM plant have to be considered in a case-specific manner, taking into account the characteristics of a particular nGM approach and the nature of the developed traits (SAM, 2017).

Certain nGMs such as reverse breeding are applicable to a limited range of plant species only and help to exploit the genetic diversity available rather than to generate genetic variability (Schaart et al., 2016). Other nGMs like genome editing can be applied very broadly to all major annual crops and forest trees, and their respective genomes can be specifically targeted to introduce a variety of different traits. At present, the range of possible new traits and the crops that can be targeted seem to be constrained mostly by the limited knowledge of functional genomics and crop biology (Scheben et al., 2017).

The level of risk associated with a certain nGM plant depends significantly, but not exclusively on the effects of the modified trait(s) on the overall characteristics of the modified plant species (Duensing et al., 2018). With regards to the effects of the modified traits the risk assessment needs to consider intended effects, as well as any unintended or unforeseen consequence of the expression of these modified trait(s). Three categories of nGM plants can be distinguished with respect to the target traits:

1. nGM plants with trait(s) which are related to traits occurring in crops produced by conventional approaches and are used without adverse effects for comparable purposes. Typically these nGM plants will not contain non-native genes or genomic changes, that are not yet present in cultivated populations of the plant species (Schaart et al., 2016). Several examples for this category are available, including herbicide resistant plants, plants with altered composition and plants resistant to e.g., fungal pathogens. The experience available with conventional plants harboring comparable traits can be used to judge whether plausible risks due to the specific traits may be expected.
2. nGM plants with traits similar to those established in GM plants, e.g., herbicide resistance, disease resistance or insecticidal traits. For this category of nGM plants similar approaches for risk assessment to those implemented for the respective GMOs should be applied. Previous experiences with the assessment of such GMOs should be taken into account for the development of risk assessment approaches specifically adapted to the characteristics of nGM plants.
3. nGM plants with traits which could not yet be established by conventional or other biotechnological methods. This category contains only novel, i.e., new and untried, traits developed by nGMs, e.g., through multiplexed approaches of genome editing resulting in complex physiological changes.

Our review of the available literature indicates that a wide range of nGM plants with novel traits is currently being developed for future agricultural use. Typically prior knowledge regarding safe use of these nGM plants is insufficient and the available information related to physiological functions of the modified genes and the effect of the specific modification(s) may be very limited.

Some of the novel traits will be based on multiple genetic modifications with possible complex impacts on metabolism and phenotype. Emerging methods, e.g., for multiplexed genome editing, simplify the rapid and simultaneous modification of multiple genome targets. Multiplexing increases the range of phenotypic changes that can be achieved at once, but also the depth (i.e., the extent) of molecular and physiological intervention. The present capacity of other biotechnological or conventional methods to achieve similar outcomes is limited. Typically no history of safe use is available for nGM applications and that increases uncertainty as to whether unintended effects may be associated with a particular application. Thus, the novelty status of traits developed with nGMs is a crucial factor regarding the risk assessment of nGM plants (HCB, 2017).

However, possible risks are not restricted to nGM plants with novel traits. Experience with either conventional HR plants or GM plants indicate that plausible risk hypotheses may also apply to many of the nGM plants currently being developed to express traits that are not novel. Two examples illustrate the range of environmental risks: (i) In the case of resistance of nGM plants to abiotic stress, e.g., drought (Zhang et al., 2016a; Shi et al., 2017a) or salinity (Duan et al., 2016), possible environmental risks related to the outcrossing of such traits into related species need to be addressed; (ii) In the case of HR nGM plants compositional changes through herbicide application as well as cocktail mixes of pesticide residues need to be assessed for food and feed safety while indirect risks related to e.g., changes in weed management need to be addressed in terms of environmental safety.

The following aspects should be considered for the case-specific framing of a risk assessment of nGM plants, no matter whether the trait is novel or known: (i) the knowledge available for the targeted genomic locus and the impact, (ii) of the (genetic) modification, and (iii) of the expression of the modified trait on the physiology and phenology of the nGM plant. Our findings indicate that very diverse cellular mechanisms and functional pathways are involved in different groups of nGM applications: (1) HR plants, (2) plants with resistance to diseases, (3) plants with changed composition, and (4) plants with increased resistance to environmental stressors and altered morphology or reproduction. Significant differences concerning relevant risk issues also exist between individual applications in those groups. The level of new information required to assess the respective issues should consider the extent of scientific knowledge and experience available for the specific nGM plants and traits.

It is doubtful, that the overall experience with traits derived from classical mutagenesis can provide a safe history of use for all novel traits developed e.g., by SDN-1 applications. It is reassuring that in the past no plant safety issues emerged for the mutants developed by classical mutagenesis (Duensing et al., 2018). However, this conclusion cannot simply be extrapolated to all SDN-1 traits, because, on the one hand, a fair number of these traits are novel, and on the other hand, adverse effects may not always be selected out during further crossbreeding steps and selection—steps which are indispensable in applications of classical mutagenesis. Without having analyzed possible effects caused by a particular genetic change a general assumption of safety for all SDN-1 applications lacks a robust scientific basis.

Novel traits may be developed in a very specific manner, e.g., by genome editing approaches. However, it should be noted that the level of specificity of an nGM approach *per se* does not provide an adequate measure of the level of risk associated with the respective trait.

On the other hand the level of specificity should be considered during the assessment of unintended effects related to the methods employed. Again, the specific characteristics of the respective nGM methods (i.e., how they work and at which stage they are used) as well as their level of specificity have to be considered in a case-specific manner. The need for such an approach is illustrated by the spectrum of available methods for genome editing, including ODM and the many different applications of the CRISPR-system. As mentioned, these methods introduce different modifications including (i) small random mutations at specific genomic loci (SDN-1), (ii) directed, but typically small sequence changes at specific genomic locations (SDN-2 and base-editing), and (iii) targeted insertion of exogenous genetic constructs and transgenes (SDN-3). In addition, specific epigenetic changes can be achieved by modifying the methylation pattern. Different levels of off-target activity and different outcomes are associated with the different approaches. Even if the number of off-target mutations may be lower for genome editing approaches compared to some approaches for random mutagenesis, especially when disregarding subsequent screening and breeding steps, they should not be neglected. A case-specific analysis of off-target activity can provide useful indications whether potential adverse outcomes may be expected (Zhao and Wolt, 2017). This approach should not just rely on predictions by bioinformatics, since these tools might not be robust enough yet (Cameron et al., 2017; Zischewski et al., 2017). Additional analytical testing is required and a range of approaches is available for focused as well as unbiased genome-wide assessment (Agapito-Tenfen et al., 2018).

Schemes to develop nGM plants typically involve a combination of different technologies. Most nGM approaches also involve GM technology at certain (intermediate) steps and/or techniques of cell and tissue cultivation and regeneration, e.g., protoplast technology, which cause an elevated level of random genetic change (Wolt, 2017). Therefore, genome editing approaches should not be solely judged by the specificity of their mechanism, e.g., the characteristics of the used type of site-directed nuclease. On the contrary, a comprehensive view is required to consider the potential of the overall development process to either induce unintended genetic changes or to remove unwanted mutations during downstream steps. Some nGMs like genome editing can speed up breeding processes significantly, e.g., by direct modification of elite lines, which in turn can impair the likelihood to detect and remove those unintended genetic changes, which are not genetically linked to the intended modification, when the final product is established.

In our opinion a general assessment framework should be implemented for nGM plants, which is addressing the characteristics of each particular nGM plant, its traits and the consequences of unintended effects. It would incorporate the following elements, some of which are recommended to be used in a case-specific way by other authors as well (Huang et al., 2016; Ricroch et al., 2016; HCB, 2017):

- Case-specific risk assessment requirements, which take into consideration the nature of the developed traits, unintended consequences of the introduced modifications, the available experience with comparable products and relevant protection goals specified by the respective countries.
- Appropriate molecular characterization, to assess among other things whether any transgenic inserts are unintentionally present in final nGM products and to determine the presence of off-target modifications and other unintended genetic changes, which might result in adverse phenotypic effects.
- Phenotypic characterization to specifically test parameters related to plausible risk issues associated with particular nGM plants, that are not covered by other existing legislation applicable to nGM plants [e.g., plant variety registration, food safety, and others (see Spranger, 2017)].

For a robust characterization of unintended effects in nGM plants we recommend that risk assessors apply a 10 step approach as proposed and outlined in Box 1. The outlined steps are based on considerations discussed in more detail throughout this study.

Box 1 **Proposal for a 10 step approach to characterize nGM plants regarding unintended effects.**

Steps 4–6 are specific for genome editing applications; the other steps are relevant for all nGM applications. For the development of concrete criteria for the risk assessment of nGM plants these points need to be further elaborated based on the emerging knowledge and state of the art of analytical methods.

1. Consider the specific characteristics of the applied nGM approach, including method particulars and the targeted plant species, to check whether it is known for a potential to induce unintended changes. This should include but not be limited to off-target activity. In the case of genome editing applications consider if the particular biotechnological process has been optimized for precision, i.e., to result in a low level of off-target activity.
2. Check, if information is available from previous use of comparable approaches which is indicating a certain potential for unintended changes/off-target activity.
3. Assess the probability that genetically unlinked unintended modifications will be removed by crossbreeding used to develop a final product. This assessment should be based on the breeding history of the final product.
4. Use robust bioinformatics tools to predict potential sites for off-target changes in the reference genome of the respective plant species, if available. In case no adequate (reference) genome sequence data is available, use a whole genome sequencing approach to check on actual off-target modifications (see point 6 below).
5. Apply the available suite of *in vitro* test methods to identify a “superset of potential off-target cleavage sites” for a particular genome editing method (Akcakaya et al., 2018). This allows to check on the quality of the bioinformatics-based prediction of potential off-target sites. Consider if *in vitro* testing identifies potential activity at sites that are non-homologous to the genomic target sequence and thus not included in the prediction by bioinformatics tools.
6. Based on the above and a wider set of potential off-target sites, use targeted sequencing to detect actual off-target changes at the predicted genomic loci. Use targeted sequencing also to assess the genomic region which is genetically linked to the desired modification(s), i.e., in the (wider) vicinity of the target sequence for the intended changes.
7. Use whole genome sequencing to scan for unintended changes in a non-biased way in case unlinked modifications have not been removed and the used method protocols are not optimized for high specificity or the method is known to be associated with off-target activity and no robust prediction of off-target activity is possible. The appropriate comparator is the genome sequence of the parental plant line which was subjected to modification by nGM approaches.
8. Assess whether any unintended changes might be of functional biological relevance. Consider if unintended changes might result in non-conservative nucleotide exchanges in coding sequences. Additionally consider whether unintended sequence changes might impact the regulatory function of the modified sequence.
9. Check whether it is possible to assess the significance of unintended changes in terms of biological effects. The following information might be helpful for such considerations: reference sequence data, further sequencing information from different plant lines to assess the degree of natural variability of a particular genomic sequence and annotations of the functions of specific genomic sequences.
10. Targeted or untargeted phenotyping should be used to assess the possibility of adverse effects resulting from unintended modifications/off-target effects. In particular such an assessment should be required in case fast-track approaches are used to develop the final product (e.g., modification of elite lines, few or no crossbreeding possible or applied following the modification step). Such assessments should also be required in the case potential phenotypic effects are associated with identified unintended sequence changes which cannot be readily removed from the final product by crossbreeding (cf. results of points 4, 8, and 9).

The existing regulatory framework in the EU for GMOs includes requirements for a scientific risk assessment conducted by EFSA (Agapito-Tenfen et al., 2018). The currently applied assessment approach is based on a case-specific problem formulation according to the principles and the general process laid out in Directive 2001/18/EC (EFSA, 2010).

The recent ruling of the European Court of Justice confirms that in the EU plants developed by genome editing approaches are covered by existing biosafety legislation, in particular Directive 2001/18/EC, and are thus subject to the requirements for a premarket risk assessment according to the comprehensive general framework outlined in the directive (ECJ, 2018). If GM technology is involved in the development process of other nGM plants, similar risk assessment requirements apply.

EFSA has already conducted an initial evaluation for some nGM applications, i.e., plants developed through cisgenesis, intragenesis, and SDN-3 type applications of genome editing, as to whether and how specific risk issues should be considered for such nGM plants (EFSA–Panel on GMOs, 2012a,b). These studies should be revisited and used as input to develop robust risk assessment approaches for such applications. Similar evaluations need to be conducted for all nGM applications included in the ruling of the ECJ, particularly for emerging technologies like CRISPR-based genome editing which can be applied in many ways and with many variants. The experience available with risk assessments for nGM products according to the existing worldwide regulatory frameworks for biosafety should be taken into account during this exercise. However, at present the experience with such assessments is quite limited (Wolt, 2017), partly due to the decisions of a number of countries not to regulate some nGM plants (Waltz, 2018). Against this background of limited knowledge and experience we recommend that a case-specific risk assessment is conducted for nGM plants to address all relevant risk issues accordingly. Our technical analysis is thus in agreement with the outcome of the ECJ ruling.

## Conclusion

A broad range of nGMs including genome editing is currently available and further methods allowing complex modification of plants are rapidly being developed. They are used to develop nGM plants with different traits and characteristics, which will be associated with different levels of risk. With respect to intended traits three categories of nGM plants can be distinguished (apart from further considerations regarding e.g., crop type, purpose of application, and use, etc., that have to be taken into account additionally):

1. nGM plants with traits and usage known from conventional approaches and without adverse effects
2. nGM plants with traits known from established GM plants, e.g., herbicide resistance or disease resistance, and associated with comparable risk issues
3. nGM plants with traits which have not yet been established and thus need to be considered as novel.

Our study shows that nGM applications may be found for all three categories; the same applies for all sub-classes of genome editing (SDN-1, SDN-2, and SDN-3). Therefore, regulation and risk assessment has to acknowledge that all nGM groups will be comprised of a mix of applications with lower as well as higher uncertainty regarding their level of risk/safety. In addition nGM applications are fairly new and only a few plants developed with these methods have been risk assessed for cultivation purposes so far. Against this background of insufficient knowledge and experience for a variety of applications, we argue that a general framework for biosafety oversight is further implemented for nGM plants, based on a case-specific risk assessment incorporating the following elements:

- Case-specific risk assessment requirements taking into account (i) the nature of the developed trait, (ii) unintended consequences of the modification introduced, (iii) the available experience with comparable products, and (iv) relevant protection goals specified by the respective countries.
- Appropriate molecular characterization to assess among other things (i) the unintentional presence of any transgenic inserts in the final product, and (ii) the presence of off-target modifications and other unintended genetic changes, which might result in adverse phenotypic effects.
- Phenotypic characterization to specifically test parameters related to plausible risk issues associated with a particular nGM plant.

This will require that the existing guidance for risk assessment of GMOs as established in the EU by EFSA be reviewed as to whether it is suitable, sufficient and appropriate for specific types of nGM applications. Specific guidance needs be developed which enables risk assessors to focus their attention and resources on issues of concern specific for the different applications and to use established and emerging tools for their assessment.

With a view to the development of ever faster and ever more complex and sophisticated breeding approaches this will not be an easy task. However, in our opinion the efforts will be worthwhile from a safety perspective and a better alternative to exempting nGM applications from biosafety assessments altogether.

## Author Contributions

ME conducted the study and drafted the manuscript. MD and MM contributed to data acquisition and analysis. AH, WR, RS, and FW contributed to the study design and implementation and edited the manuscript. All authors read and approved the manuscript for publication.

### Conflict of Interest Statement

The authors declare that the research was conducted in the absence of any commercial or financial relationships that could be construed as a potential conflict of interest.
