# Corrigendum: An EU Perspective on Biosafety Considerations for Plants Developed by Genome Editing and Other New Genetic Modification Techniques (nGMs)

**DOI:** 10.3389/fbioe.2019.00090

**Published:** 2019-05-01

**Authors:** Michael F. Eckerstorfer, Marion Dolezel, Andreas Heissenberger, Marianne Miklau, Wolfram Reichenbecher, Ricarda A. Steinbrecher, Friedrich Waßmann

**Affiliations:** ^1^Department Landuse & Biosafety, Environment Agency Austria, Vienna, Austria; ^2^Department GMO Regulation, Biosafety, Federal Agency for Nature Conservation, Bonn, Germany; ^3^EcoNexus, Oxford, United Kingdom

**Keywords:** new genetic modification techniques (nGM), genome editing, CRISPR/Cas, plant modification, biosafety, risk assessment

Marion Dolezel and Marianne Miklau were not included as authors in the published article. The corrected Author Contributions Statement appears below. The authors apologize for this error and state that this does not change the scientific conclusions of the article in any way. The original article has been updated.

## Author Contributions

ME conducted the study and drafted the manuscript. MD and MM contributed to data acquisition and analysis. AH, WR, RS, and FW contributed to the study design and implementation and edited the manuscript. All authors read and approved the manuscript for publication.

